# Efficacy of focused ultrasound for HPV clearance and cervical LSIL treatment: a meta-analysis

**DOI:** 10.1038/s41598-026-45421-4

**Published:** 2026-03-28

**Authors:** Jiangwei Luo, Yong Lin, Qinqin Yi, Yang Long

**Affiliations:** Luzhou Maternal and Child Health Hospital (Luzhou Second People’s Hospital), Luzhou city, Sichuan Province China

**Keywords:** Focused ultrasound, HPV, LSIL, Cervical cytology, Meta-analysis, Signs and symptoms, Disease prevention, Public health

## Abstract

Persistent high-risk human papillomavirus (hrHPV) infection is a major risk factor for high-grade squamous intraepithelial lesions (HSIL) and cervical cancer. Although HPV vaccines effectively prevent infections with vaccine-covered HPV types, they do not eliminate established infections. Additionally, not all HPV types associated with cervical cancer are covered by the vaccine. Therefore, treatment strategies for HPV-related cervical lesions remain an important clinical challenge. A systematic search was conducted in PubMed, EMBASE, Web of Science, and the Cochrane Library to identify studies evaluating the efficacy of focused ultrasound in treating HPV and cervical low-grade squamous intraepithelial lesions (LSIL). Ten eligible observational studies were included. Study quality was assessed using the MINORS criteria, and evidence quality was evaluated based on GRADE guidelines. A meta-analysis was performed using Stata 12.0 software. Focused ultrasound treatment led to HPV clearance in 74% of cases (ES = 0.74, 95% CI: 0.64–0.85, *P* < 0.001). Additionally, 94% of women with LSIL histology before treatment had a normal cervical biopsy at follow-up (ES = 0.94, 95% CI: 0.92–0.97, *P* < 0.001), and 87% of women with abnormal ThinPrep cytology (TCT) results had normal cytology at follow-up (ES = 0.87, 95% CI: 0.78–0.96, *P* < 0.001). Compared to the observation group, focused ultrasound treatment was significantly more effective in clearing HPV (OR = 3.58, 95% CI: 2.21–5.81, *P* < 0.001). Similarly, focused ultrasound was superior to interferon treatment for HPV clearance (OR = 4.22, 95% CI: 1.12–15.96, *P* = 0.034). The quality of evidence across studies was rated as low to moderate. This meta-analysis demonstrates that focused ultrasound achieves a 74% HPV clearance rate and 94% LSIL resolution in women with cervical LSIL and concurrent hrHPV infection. While superior to observation and interferon, the evidence remains low-to-moderate due to the observational nature and geographic concentration of included studies. Future multicenter RCTs are essential to validate these results and assess long-term outcomes, including recurrence and obstetric safety.

**Supplementary Information:**

The online version contains supplementary material available at 10.1038/s41598-026-45421-4.

## Introduction

Cervical cancer, predominantly caused by the human papillomavirus (HPV), remains a major global health challenge. HPV is critically implicated in the development and progression of cervical cancer. According to recent estimates, there were 662,044 new cases of cervical cancer worldwide in 2022, with an age-standardized incidence rate (ASIR) of 14.12 per 100,000 women. This disease also led to 348,709 deaths, making it the fourth leading cause of cancer-related morbidity and mortality among women globally^1,2^.

Human papillomavirus replication in dividing epithelial cells is accompanied by increased expression of E6 and E7 oncoproteins. These oncoproteins are responsible for genomic instability, cell cycle disruption, cell proliferation, immortalization, and malignant transformation of HPV-infected cells. In addition, E6 and E7 oncoproteins induce immunosuppression, which prevents the immune system from detecting HPV-infected and transformed cells. Integration of HPV into the host cell genome results in the upregulation of E6 and E7 expression, contributing to HPV-associated cervical cancer^3^.

Human papillomavirus (HPV) vaccines can prevent precancerous lesions and cancer. A study has shown that the tetravalent HPV vaccine is effective in preventing HPV infection and shows a trend of sustained protection for 14 years after vaccination^4^. In Denmark, a nationwide cohort study reported an 86% reduction in cervical cancer among individuals vaccinated at age 16 or younger, and a 68% reduction among older teenagers^5^. A Swedish study observed a 62% decrease in cervical cancer incidence among women vaccinated between the ages of 20 and 30^6^. In the UK, the vaccine efficacy varied by age at vaccination; a 34% reduction in cervical cancer incidence was noted among those vaccinated at ages 16 to 18, a 62% reduction for those aged 14 to 16, and a 90% reduction for those who were vaccinated at ages 12 to 13^7^. These findings underscore the critical impact of early vaccination on reducing the risk of cervical cancer.

However, when the cervix is already infected with HPV, the HPV vaccine has limited effect in treating the HPV virus^8^. Vaccination and screening coverage rates remain low worldwide for a variety of reasons^9,10^. The treatment of HPV and the prevention of cervical lesions remain important tasks. If HPV is not treated in a timely manner, especially high-risk HPV or persistent HPV such as HPV16, HPV18, the risk of CIN2 + and even cervical cancer is significantly increased^11,12^.

There are various ways to clear HPV, such as drug therapy like Nocardia rubra cell wall skeleton immunotherapy, interferon, etc^13,14^. Focused ultrasound and LEEP treatment can also address cervical HPV^15^. Focused ultrasound is a novel method for the treatment of cervical intraepithelial neoplasia (CIN). Using the focused ultrasound beam to target tissues, Focused Ultrasound surgery (FU) induces thermal, mechanical, and cavitation effects. Unlike other ablation therapies, FU offers good permeability and different treatment modes (inside-out). Degeneration of the cervical tissue 3 to 6 mm deep and on the surface of the cervix occurs. This process can promote necrosis and enable the affected cells to be replaced by surrounding normal tissue^16^. Focused ultrasound (FU) is a non-invasive thermal ablation technique that utilizes high-intensity focused sound waves to generate localized heat (typically 60–100 °C), inducing coagulative necrosis in targeted cervical tissue up to 3–6 mm deep^16^. Unlike LEEP, which excises tissue, FU promotes in situ degeneration and subsequent replacement by surrounding normal epithelium, preserving cervical architecture—making it particularly suitable for women of reproductive age^16^. It is crucial to note that FU does not act as an antiviral agent; rather, it removes HPV-infected epithelial cells, thereby facilitating immune-mediated viral clearance.

Although most low-grade lesions do not require treatment, 10% of low-grade lesions become high-grade. Furthermore, due to the stress following LSIL diagnosis and persistent infection with papillomavirus, especially high-risk HPV, some patients will seek medical intervention, particularly those who want to have children^17^. Until now, there has been no systematic review to evaluate the efficacy and safety of focused ultrasound in the treatment of HPV and low-grade squamous intraepithelial lesions (LSIL). Therefore, we conducted a meta-analysis to systematically evaluate the efficacy of focused ultrasound in the treatment of HPV and LSIL. The aim is to establish a reliable protocol for young women and assist clinicians in the management of HPV.

## Materials and methods

### Inclusion and exclusion criteria

Inclusion criteria: (1) Study designs included observational studies (cohort, case-control), clinical trials, and comparative studies. Randomized controlled trials (RCTs) were eligible but none were identified. (2) Participants: adult women with histologically confirmed LSIL (CIN1) and concurrent high-risk HPV (hrHPV) infection. (3) All subjects underwent p16INK4a immunohistochemistry to confirm HPV oncogenic activity. (4) Primary outcome: HPV clearance rate at 3–6 months post-treatment.

Exclusion criteria: (1) Animal studies, cell studies, reviews, meta-analyses, case reports, or letters. (2) Studies reporting duplicated data. (3) Studies with unusable data errors. (4) Studies with non-conforming outcome measures or significant errors or omissions. (5) Studies involving women diagnosed with HSIL.

### Search strategy

To conduct a systematic research review, computer searches and manual searches were performed in PubMed, Embase, Web of Science, and the Cochrane Library databases. The search was conducted up to May 2024. The language is limited to English, regardless of the publication year. Search terms included “HPV” and “focused ultrasound”. Two researchers independently conducted computer searches and manual searches of the literature, and a third researcher was consulted in case of disagreement.

### Literature data extraction

After searching the literature, two researchers (Lin Yong and Long Yan) rigorously screened the literature based on the inclusion and exclusion criteria, extracted the data, and finally summarized the findings independently before reaching a consensus. After screening, literature information was extracted, including: (1) the first author and the publication date; (2) age and total sample size; (3) follow-up time; and (4) outcome indicators.

### Literature quality assessment

Two researchers independently used the Methodological Index for Non-Randomized Studies (MINORS) to evaluate the quality of the studies. In cases where data were missing or more details were required, we attempted to collect the necessary information by contacting the first author. In case of disagreement, the decision was made by two researchers or by a third researcher. The Methodological Index for Non-Randomized Studies (MINORS) is a quality assessment tool that can be used to evaluate observational studies, non-randomized controlled trials (non-RCT), and other literature. There were 12 evaluation indicators, each scored from 0 to 2. A score of 0 indicated no report, 1 indicated an insufficient report, and 2 indicated a sufficient report. The first eight items were for studies without a control group, with the highest score being 16. Studies with a score higher than 11 were considered high-quality literature. The last four items, along with the first eight items, were used for studies with a control group. The highest score was 24 points, and studies with a score of more than 17 points were considered high-quality literature^18^.

### Statistical analysis

Statistical analyses were conducted using Stata 12.0 to evaluate the effectiveness of focused ultrasound in achieving HPV, LSIL, and abnormal TCT clearance. The comparison also included the efficacy of focused ultrasound versus interferon treatment and versus an observational control group. Effect sizes (ES) or odds ratios (OR) along with their 95% confidence intervals (95% CI) were calculated to assess the significance of the results. A result was considered statistically significant if the 95% CI did not include 1 (for OR) or 0 (for ES), or if *p* < 0.05. Cochran’s Q test and the I^2^ statistic were used to evaluate heterogeneity among the included studies; significant heterogeneity was considered present if *p* < 0.1 and I^2^ > 50%. To assess publication bias, funnel plots, Begg’s test, and Egger’s test were employed, with *p* < 0.05 indicating potential bias.

### Risk of bias and sensitivity analysis

Publication bias has been a problem in evidence synthesis, with researchers subjectively assessing publication bias by observing the symmetry of the funnel plot. If the funnel plot is symmetrical, there is no publication bias. In addition, we also used the Begg’s test and the Egger’s test to identify publication bias in the literature, thereby complementing the subjective assessment. *P* > 0.05 indicates that the funnel plot is symmetrical, suggesting no publication bias. Sensitivity analysis was conducted by excluding each study individually, with subgroup analysis conducted where necessary to investigate the impact of different study characteristics on the observed effects and to identify potential sources of heterogeneity.

### Evaluation of the evidence quality process

Two researchers assessed the certainty of evidence using the GRADE approach. For the randomized controlled studies, we assessed the quality of evidence for each outcome based on the risk of bias, inconsistency, indirectness, publication bias, and imprecision. For observational studies, we assessed the quality of evidence for each outcome by considering the effect value, the dose-effect relationship, and the potential for bias. Two researchers will conduct the evaluation independently. If there is any dispute, the third researcher will discuss and reach an agreement. The quality of evidence is categorized into four levels: high, medium, low, and very low^19,20^.

### Ethical considerations

All analyses were based on published studies and did not require ethical approval or patient consent.

## Results

### Procedure of study retrieval and characteristics of the study

**Fig. 1:**
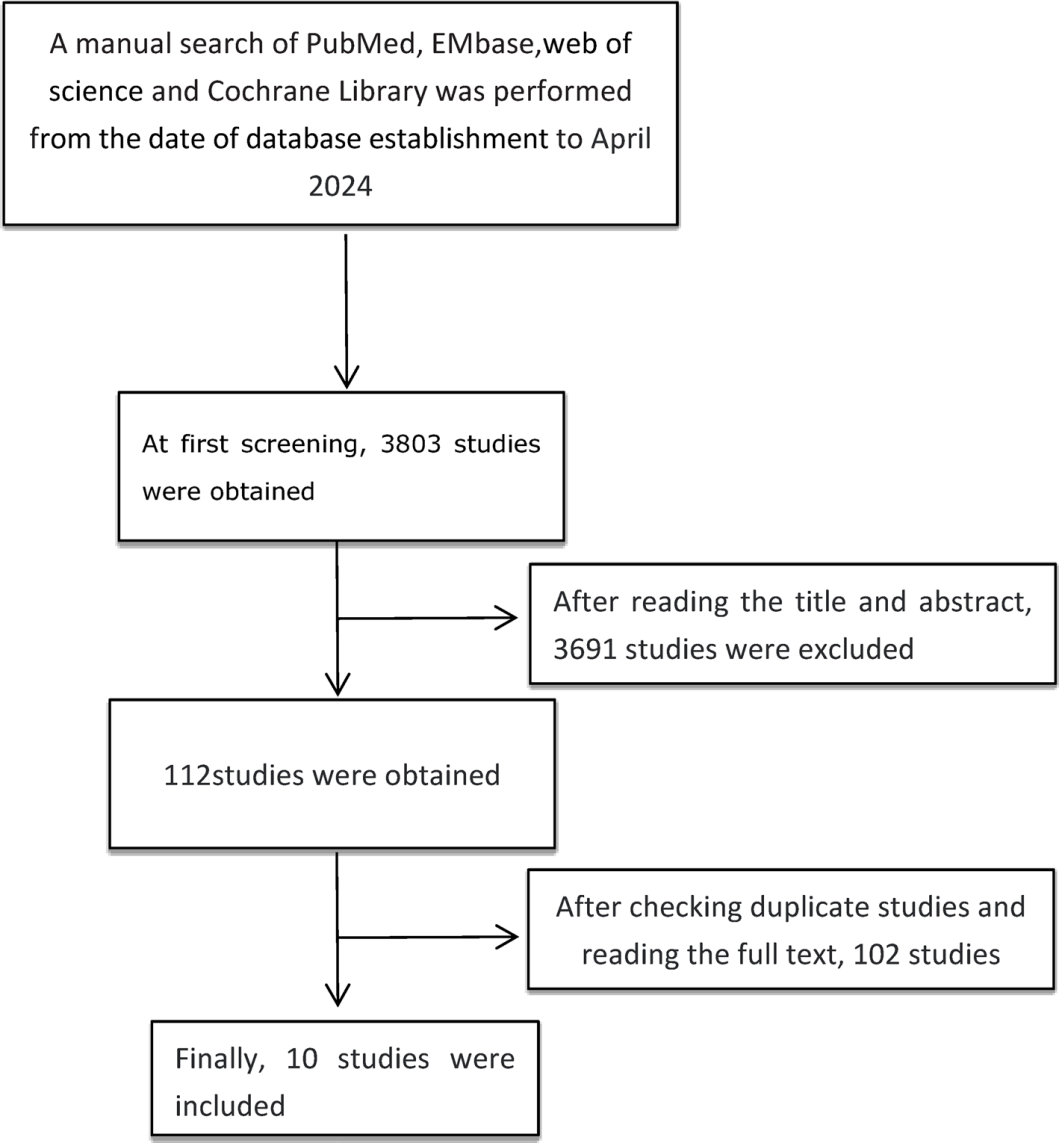
The retrieval process of the studies.

The initial search produced 3803 studies. After reviewing titles and abstracts, 112 articles remained. After checking for repeated studies and reading the full text, 10 studies were excluded, and ten studies were finally included in the meta-analysis^21–30^. The retrieval process of the studies is shown in Fig. 1. The basic characteristics of the included studies are shown in Table 1.

**Table 1:** The basic characteristics of the included studies.

Study	Intervening measure		Age (year)		Sample size	Follow-up time	Outcome
	Experimental group	Control group	Experimental group	Control group	Experimental/ control group		
C-Z Li 2009	Focused ultrasound		40.85 ± 6.61		20	Six months after treatment	HPV negative rate, LSIL negative rate, TCT negative rate
Zhenhua Fu 2020	focused ultrasound		38 ± 9.9		30	three months after treatment	HPV negative rate, LSIL negative rate, TCT negative rate
Wenping Wang 2021	Focused ultrasound	Interferon	40.25 ± 8.94	41.19 ± 9.35	300/292	Sx months after treatment	HPV negative rate, comparison of focused ultrasound and interferon for treating HPV
Hongmin Zeng 2022	Focused ultrasound		20–57			Six months after treatment	HPV negative rate
Wenping Wang 2022	Focused ultrasound	Observation group	39.1 ± 8.6	39.2 ± 8.5	86/78	Six months after treatment	HPV negative rate, comparison of focused ultrasound and observation group for treating HPV
Miaomiao He 2023	Focused ultrasound	Interferon	27.7 ± 5.8	39.6 ± 10.7	47/57	Six months after treatment	HPV negative rate, comparison of focused ultrasound and interferon for treating HPV
	Focused ultrasound	Observation group		41.9 ± 11.1	57	Six months after treatment	HPV negative rate, comparison of focused ultrasound and interferon for treating HPV
Hua Tao 2023	Focused ultrasound		18-45		169	Three months after treatment	HPV negative rate
Wenping Wang 2023	Focused ultrasound		40.4 ± 7.2		35	Three months after treatment	HPV negative rate
Wenping Wang 2023	Focused ultrasound		40.6 ± 6.7		32	Three months after treatment	HPV negative rate
Rong Tan 2009	Focused ultrasound		Over 18 years old		117	Six months after treatment	HPV negative rate, LSIL negative rate

### Assessment of bias risk of included studies

The MINORS scores of the ten studies ranged from 14 to 22. All studies used the standard instrument MINORS, and the quality scores of the included studies ranged from 14 to 22 according to the quality assessment checklist of MINORS. All studies were of high quality (Table 2).

**Table 2:** MINORS scores of the studies.

study	I	II	III	IV	V	VI	VII	VIII	IX	X	XI	XII	1. Total
C-Z Li 2009	2	2	2	2	2	1	2	1					14
Zhenhua Fu 2020	2	2	2	2	2	1	2	1					14
Wenping Wang 2021	2	2	2	2	2	1	2	1	2	2	2	2	22
Hongmin Zeng 2022	2	2	2	2	2	2	2	1					15
Wenping Wang 2022	2	2	2	2	2	2	2	1					15
Miaomiao He 2023	2	2	2	2	2	1	2	1					14
Hua Tao 2023	2	2	2	2	2	1	2	1					14
Wenping Wang 2023	2	2	2	2	2	1	2	1					14
Wenping Wang 2023	2	2	2	2	2	2	2	1					15
Rong Tan 2009	2	2	2	2	2	2	2	1					15

Note: I whether the study’s purpose is clear, II the consistency of patients included, III whether the expected data has been collected, IV whether the endpoint indicators appropriately reflect the purpose of the study, V the objectivity of the evaluation of the endpoint indicators, VI the adequacy of follow-up, VII whether the study dropout rate is less than 5%, VIII and whether the sample size has been estimated.

### Assessment of evidence quality level

#### Clearance rate of cervical HPV following focused ultrasound treatment

Ten observational studies were included in this meta-analysis. The effect size of nine studies was large, while one study showed a small effect size without significant confounding factors. As a result, the evidence quality of these nine studies was upgraded by one level. The evidence of focused ultrasound for clearing cervical HPV is of intermediate and low quality. The results are shown in Table 3.

**Table 3:** The quality of the evidence about the studies.

Study	Outcome	Effect size	Dose-effect relationship	Negative offset	Grade of evidence
C-Z Li 2009	Clearance rate of cervical HPV	Upgraded by one level	Not upgrade	Not upgrade	Intermediate
	Clearance rate of abnormal TCT	Upgraded by one level	Not upgrade	Not upgrade	Intermediate
Zhenhua Fu 2020	Clearance rate of cervical HPV	Upgraded by one level	Not upgrade	Not upgrade	Intermediate
Wenping Wang 2021	Resolution rate of LSIL	Not upgrade	Not upgrade	Not upgrade	Low
	Clearance rate of abnormal TCT	Upgraded by one level	Not upgrade	Not upgrade	Intermediate
	Clearance rate of cervical HPV	Not upgrade	Not upgrade	Not upgrade	Low
Hongmin Zeng 2022	Comparison of focused ultrasound and interferon for treating HPV	Upgraded by one level	Not upgrade	Not upgrade	Intermediate
	Clearance rate of cervical HPV	Upgraded by one level	Not upgrade	Not upgrade	Intermediate
Wenping Wang 2022	Clearance rate of cervical HPV	Upgraded by one level	Not upgrade	Not upgrade	Intermediate
Miaomiao He 2023	Resolution rate of LSIL	Upgraded by one level	Not upgrade	Not upgrade	Intermediate
	Comparison of focused ultrasound and observation group for treating HPV	Not upgrade	Not upgrade	Not upgrade	Low
	Comparison of focused ultrasound and interferon for treating HPV	Upgraded by one level	Not upgrade	Not upgrade	Intermediate
	Clearance rate of cervical HPV	Upgraded by one level	Not upgrade	Not upgrade	Intermediate
Hua Tao 2023	Clearance rate of cervical HPV	Upgraded by one level	Not upgrade	Not upgrade	Intermediate
Wenping Wang 2023	Clearance rate of cervical HPV	Upgraded by one level	Not upgrade	Not upgrade	Intermediate
Wenping Wang 2023	Clearance rate of cervical HPV	Upgraded by one level	Not upgrade	Not upgrade	Intermediate
Rong Tan 2022	Clearance rate of cervical HPV	Upgraded by one level	Not upgrade	Not upgrade	Intermediate
	Resolution rate of LSIL	Upgraded by one level	Not upgrade	Not upgrade	Intermediate

#### Resolution rate of LSIL following focused ultrasound treatment

A total of four studies were included in this meta-analysis. The effect size of three studies was large, and the effect size of one study was small without obvious confounding factors. Therefore, the evidence quality level of these three studies was upgraded by one level. Therefore, the quality level of evidence for focused ultrasound resolving cervical LSIL is considered to be intermediate and low. The results are shown in Table 3.

#### Clearance rate of abnormal TCT following focused ultrasound treatment

Two studies were included in this meta-analysis. The effect sizes of these two studies were large, and there were no significant confounding factors. Therefore, the evidence quality level of each study was upgraded by one level. Therefore, the quality of evidence for focused ultrasound in clearing abnormal TCT was intermediate. The results are shown in Table 3.

#### Comparison of HPV clearance rates between focused ultrasound and observation groups

Two studies were included in this meta-analysis. One of the studies showed a strong association between HPV clearance rates and focused ultrasound, without significant confounding factors, thereby elevating the quality of evidence in this study. The quality of the evidence was therefore low to intermediate between the focused ultrasound group and the observation group. The results are shown in Table 3.

#### Comparison of HPV clearance rates between focused ultrasound and interferon groups

The meta-analysis includes two studies. One study demonstrated a strong association between HPV clearance rates and focused ultrasound, without significant confounding factors. The quality of the evidence for the relationship between focused ultrasound and interferon was rated as low to intermediate. The results are shown in Table 3.

### Results of meta-analysis

#### Clearance rate of cervical HPV following focused ultrasound treatment

**Fig. 2:**
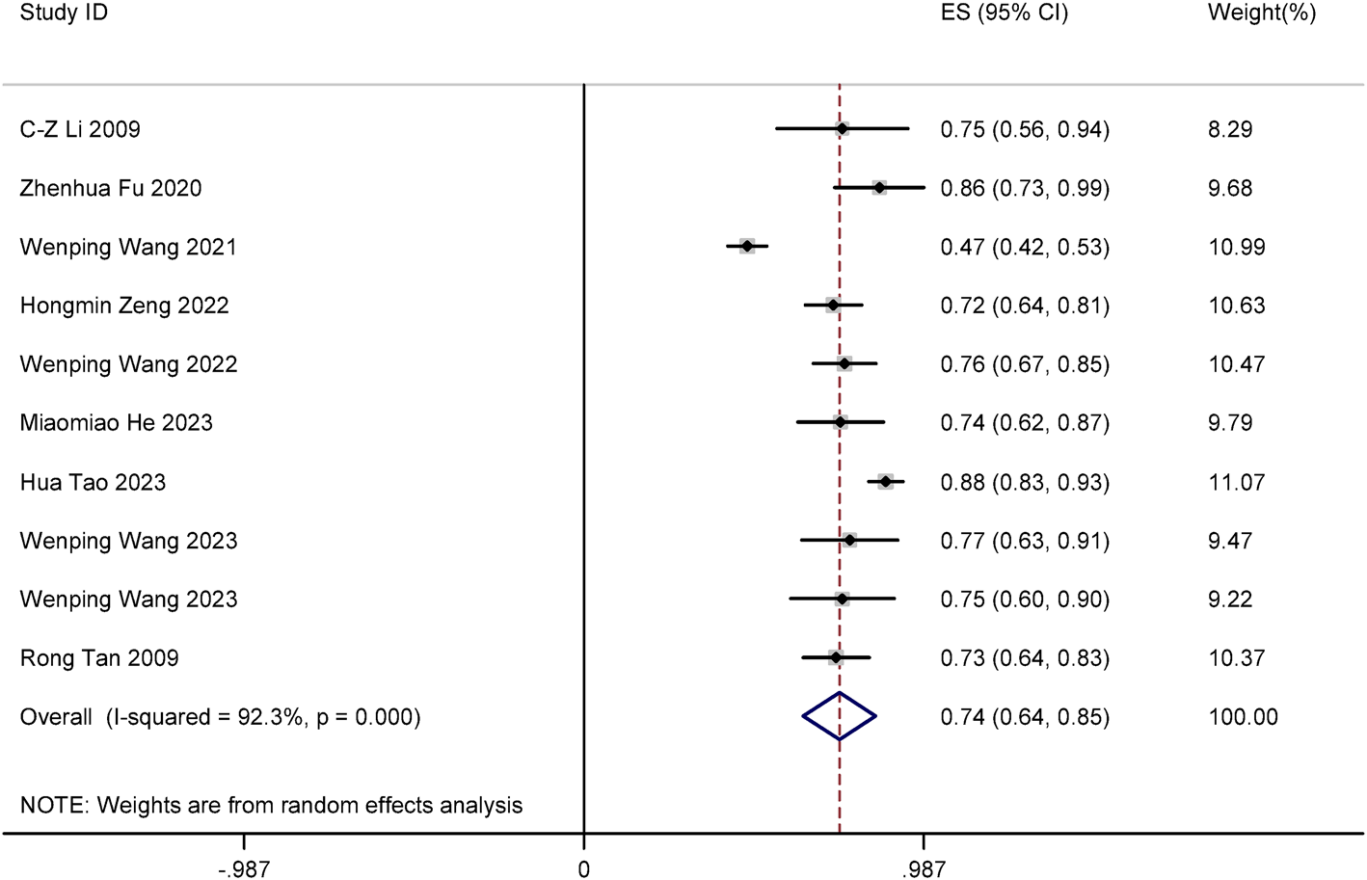
Forest plot of clearance rate of cervical HPV after focused ultrasound treatment.

The meta-analysis included 10 studies. We determined the clearance rate of HPV after focused ultrasound treatment. Significant heterogeneity was observed (I² = 92.3%, *P* < 0.001), warranting use of a random-effects model. The results showed that the clearance rate of cervical HPV after focused ultrasound treatment was 74% (ES = 0.74, 95% CI: 0.64–0.85, *P* < 0.001). The difference was statistically significant (*P* < 0.05). The forest plot is shown in Fig. 2.

#### Resolution rate of LSIL following focused ultrasound treatment

**Fig. 3:**
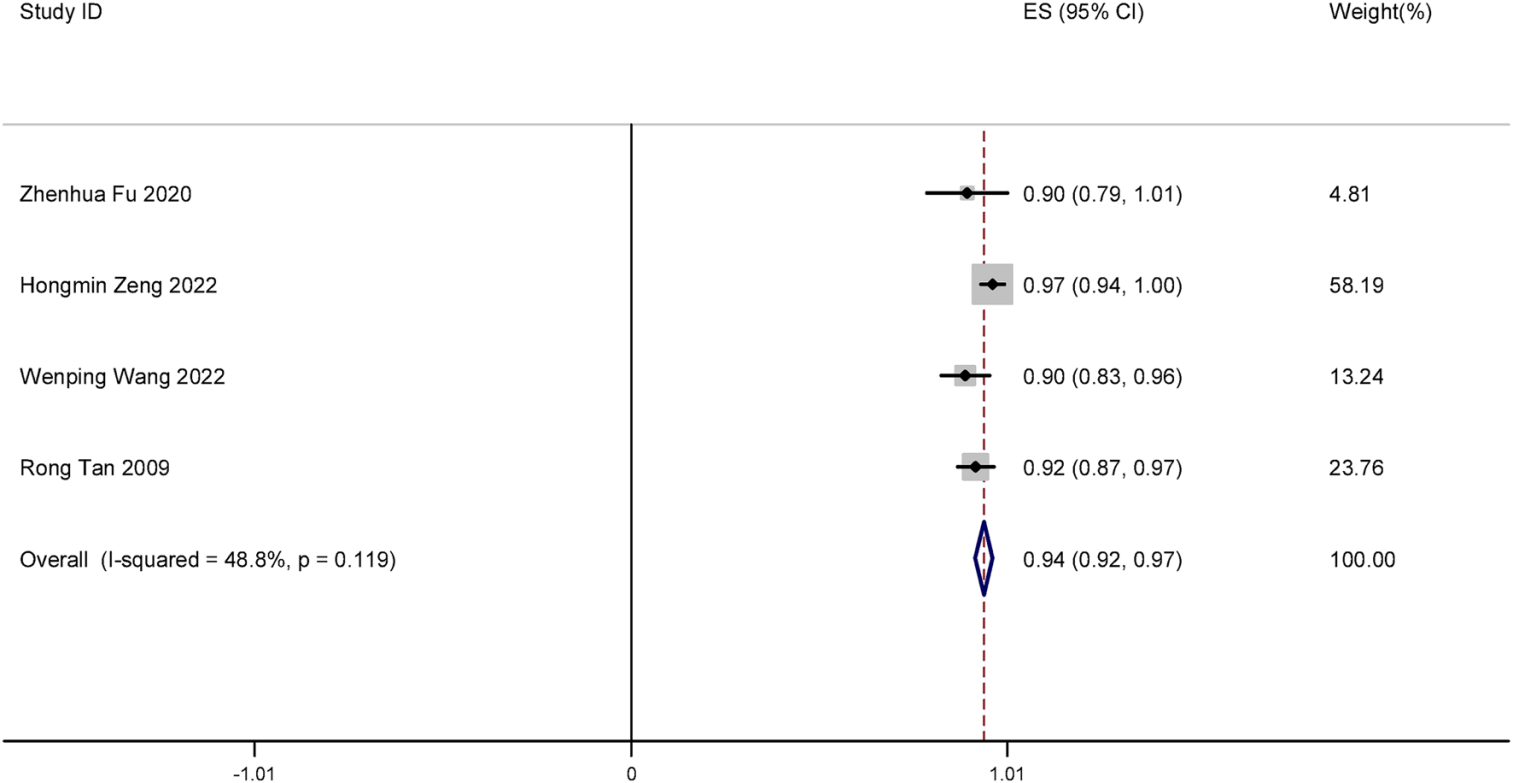
Forest plot of resolution rate of LSIL after focused ultrasound treatment for cervical LSIL.

A total of four studies were included in this meta-analysis. We determined the resolution rate of LSIL after focused ultrasound treatment. The results showed that after focused ultrasound treatment, the resolution rate of LSIL was 94% (ES = 0.94, 95% CI: 0.92–0.97, *P* < 0.001). The difference was statistically significant (*P* < 0.05). The forest plot is shown in Fig. 3.

#### Clearance rate of abnormal TCT following focused ultrasound treatment

**Fig. 4:**
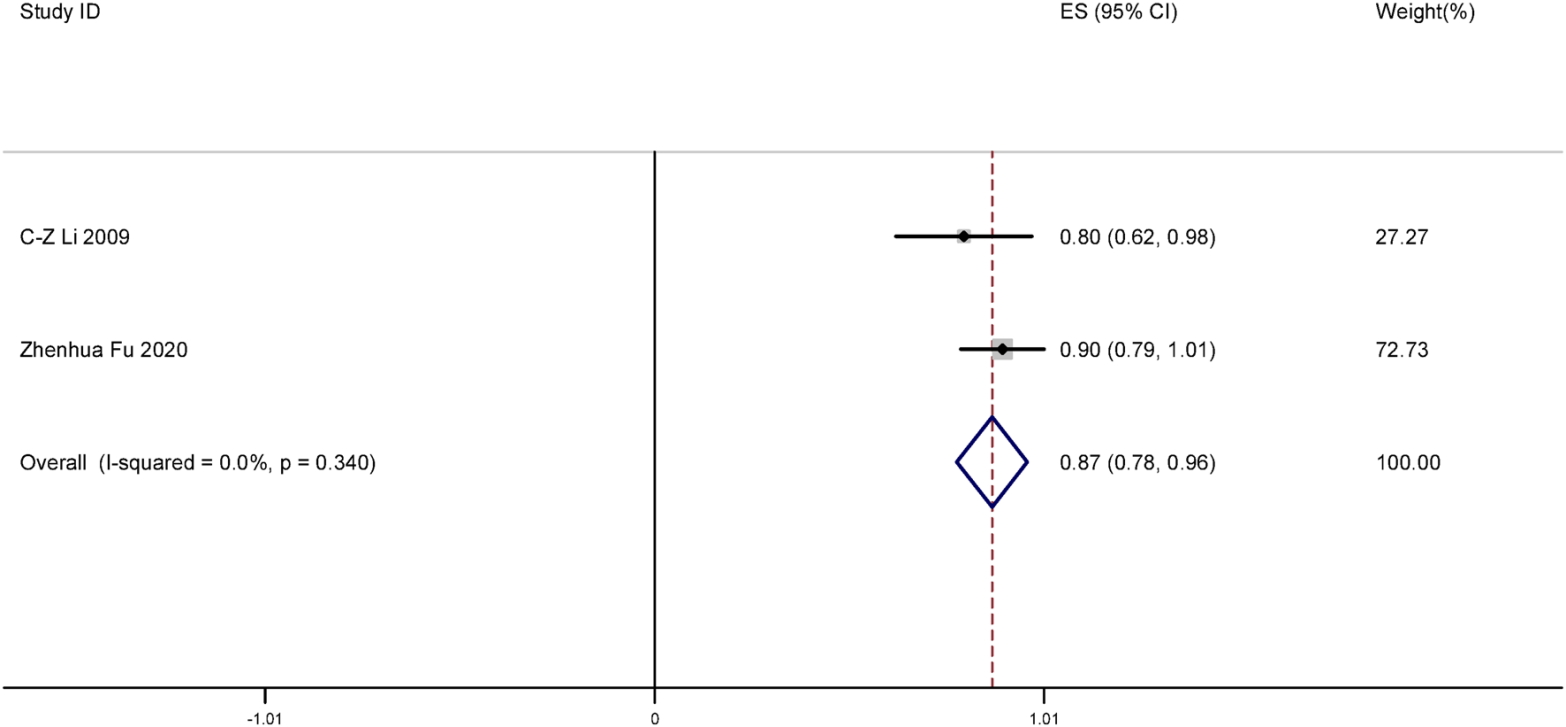
Forest plot of clearance rate of abnormal TCT after focused ultrasound clearing abnormal TCT of the cervix.

Two studies were included in this meta-analysis. We determined the clearance rate of abnormal TCT after cervical focused ultrasound clearing abnormal TCT. The results showed that after focused ultrasound clearing abnormal TCT, the clearance rate of abnormal TCT was 87% (ES = 0.87, 95% CI: 0.78–0.96, *P* < 0.001). The difference was statistically significant (*P* < 0.05). The forest plot is shown in Fig. 4.

#### Comparison of HPV clearance rates between focused ultrasound and observation groups

**Fig. 5:**
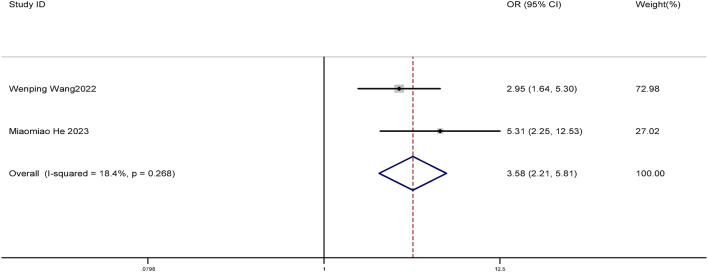
Forest plot of difference in HPV clearance rate between the focused ultrasound group and the observation group.

Two studies were included in this meta-analysis. We compared the clearance rate of HPV between the focused ultrasound group and the observation group. The results showed that the clearance rate of HPV after focused ultrasound treatment was significantly higher (OR = 3.58, 95% CI: 2.21–5.81, *P* < 0.001). The difference was statistically significant (*P* < 0.05). The forest plot is shown in Fig. 5.

#### Comparison of HPV clearance rates between focused ultrasound and interferon groups

**Fig. 6:**
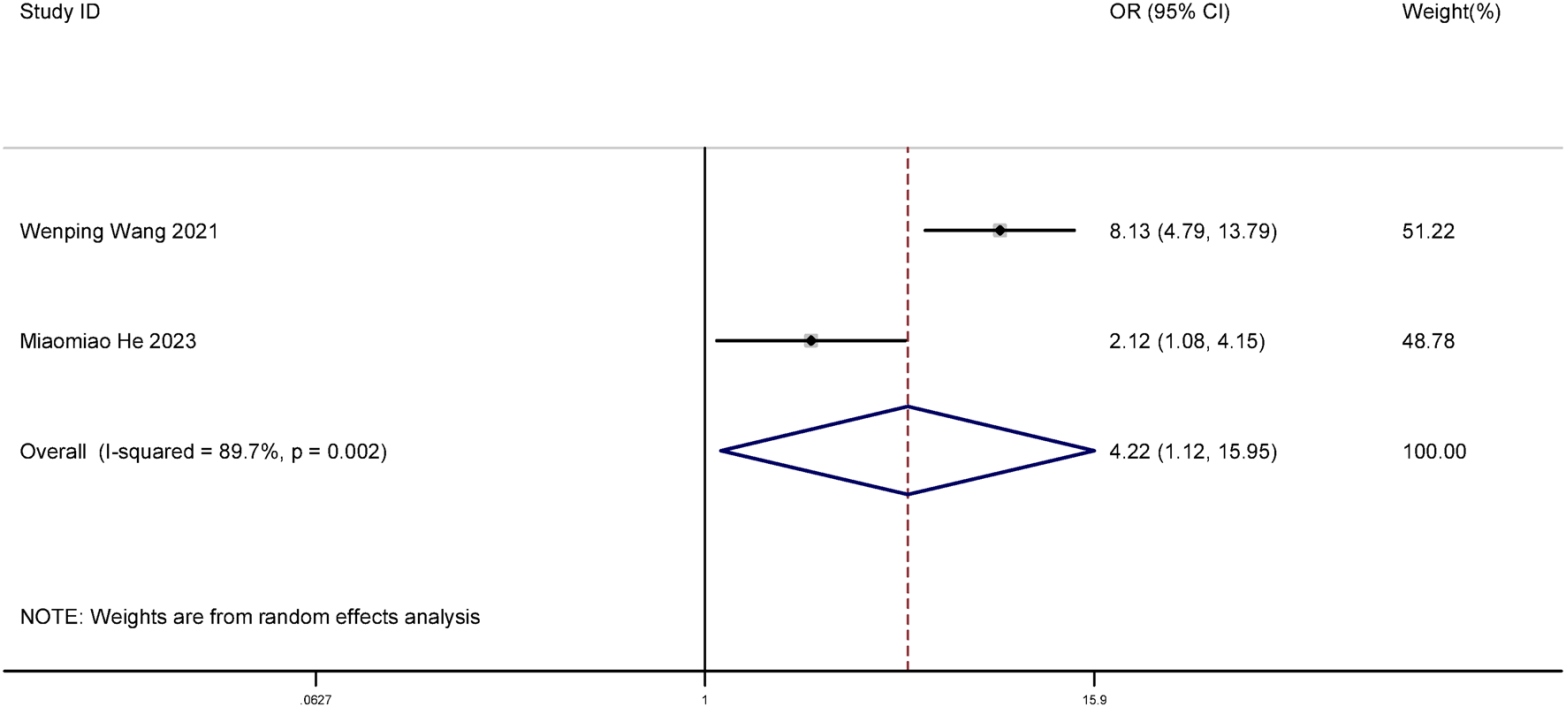
Forest plot difference in the clearance rate of HPV between the focused ultrasound group and interferon group.

Two studies were included in this meta-analysis. We compared the difference in the clearance rate of HPV between the focused ultrasound group and the interferon group. The results showed that the clearance rate of HPV after focused ultrasound treatment was higher (OR = 4.22, 95% CI: 1.12–15.96, *P* = 0.034), and the difference was statistically significant (*P* < 0.05). The forest plot is shown in Fig. 6.

### Sensitivity analysis

**Fig. 7:**
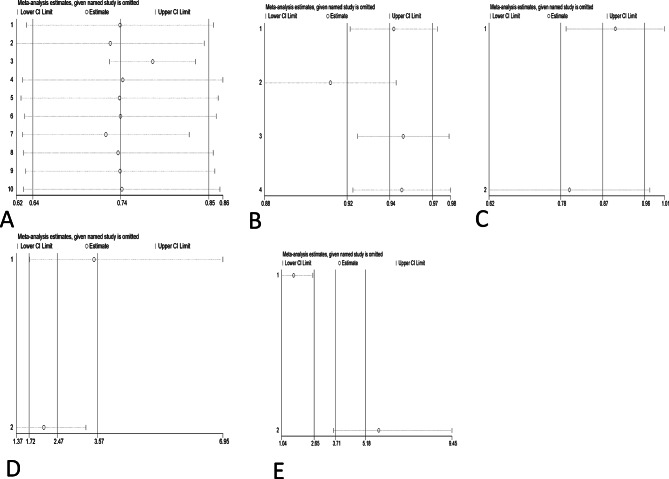
Sensitivity analysis. A Clearance rate of cervical HPV after focused ultrasound treatment. B Resolution rate of LSIL after focused ultrasound treatment of cervical LSIL. C Clearance rate of abnormal TCT after focused ultrasound treatment for abnormal TCT of the cervix. trimming method: before and after iteration, both fixed effect model and random effect model are meaningful, and the results are robust. D Difference in HPV clearance rate between the focused ultrasound group and the observation group. E Difference in the clearance rate of HPV between the focused ultrasound group and interferon group.trimming method: before and after iteration, both fixed effect model and random effect model are meaningful, and the results are robust.

Sensitivity analysis was conducted by systematically removing one study at a time to evaluate its influence on the aggregated results. Combined with the subtraction analysis, the results showed that the outcome of each meta-analysis with a 95% confidence interval was not significantly influenced by any of the individual studies. This suggests that the results of this meta-analysis are relatively reliable overall. The results of the sensitivity analysis are shown in Fig. 7.

### Publication bias

**Fig. 8:**
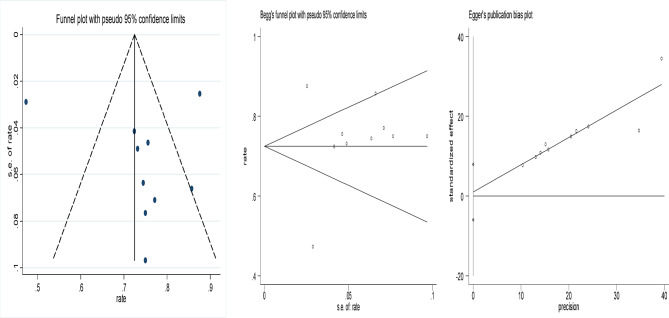
Funnel plot of clearance rate of cervical HPV after focused ultrasound treatment. Begg’s Test *p* = 0.858, Egger’s Test *p* = 0.751.

**Fig. 9:**
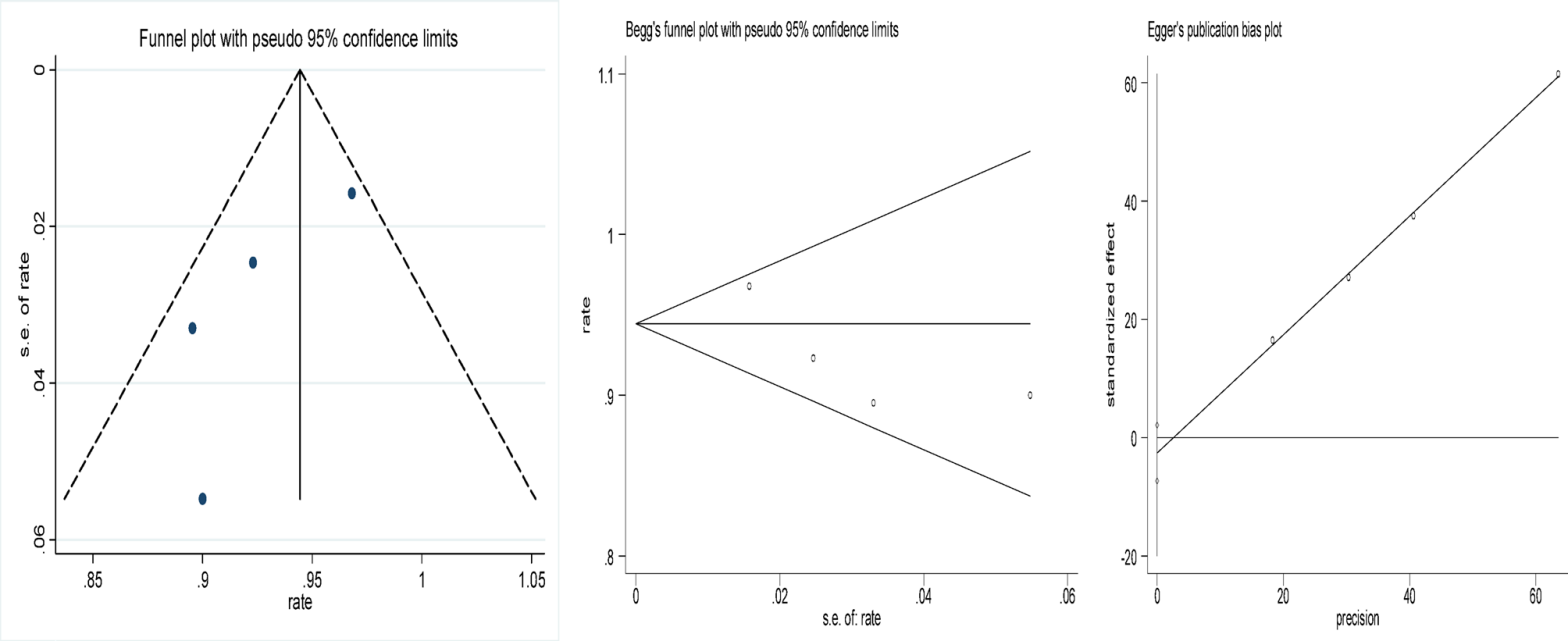
Funnel plot of resolution rate of LSIL after focused ultrasound treatment for cervical LSIL. Begg’s Test *p* = 0.734, Egger’s Test *p* = 0.137.

**Fig. 10:**
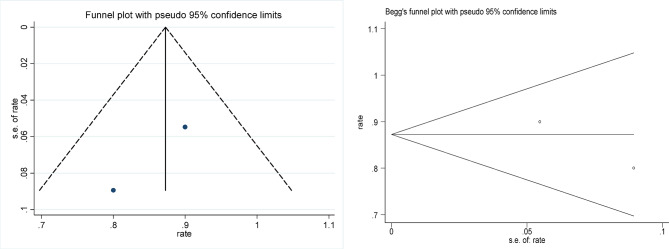
Funnel plot of clearance rate of TCT after focused ultrasound clearing abnormal TCT of the cervix. Begg’s Test *p* = 1.000.

**Fig. 11:**
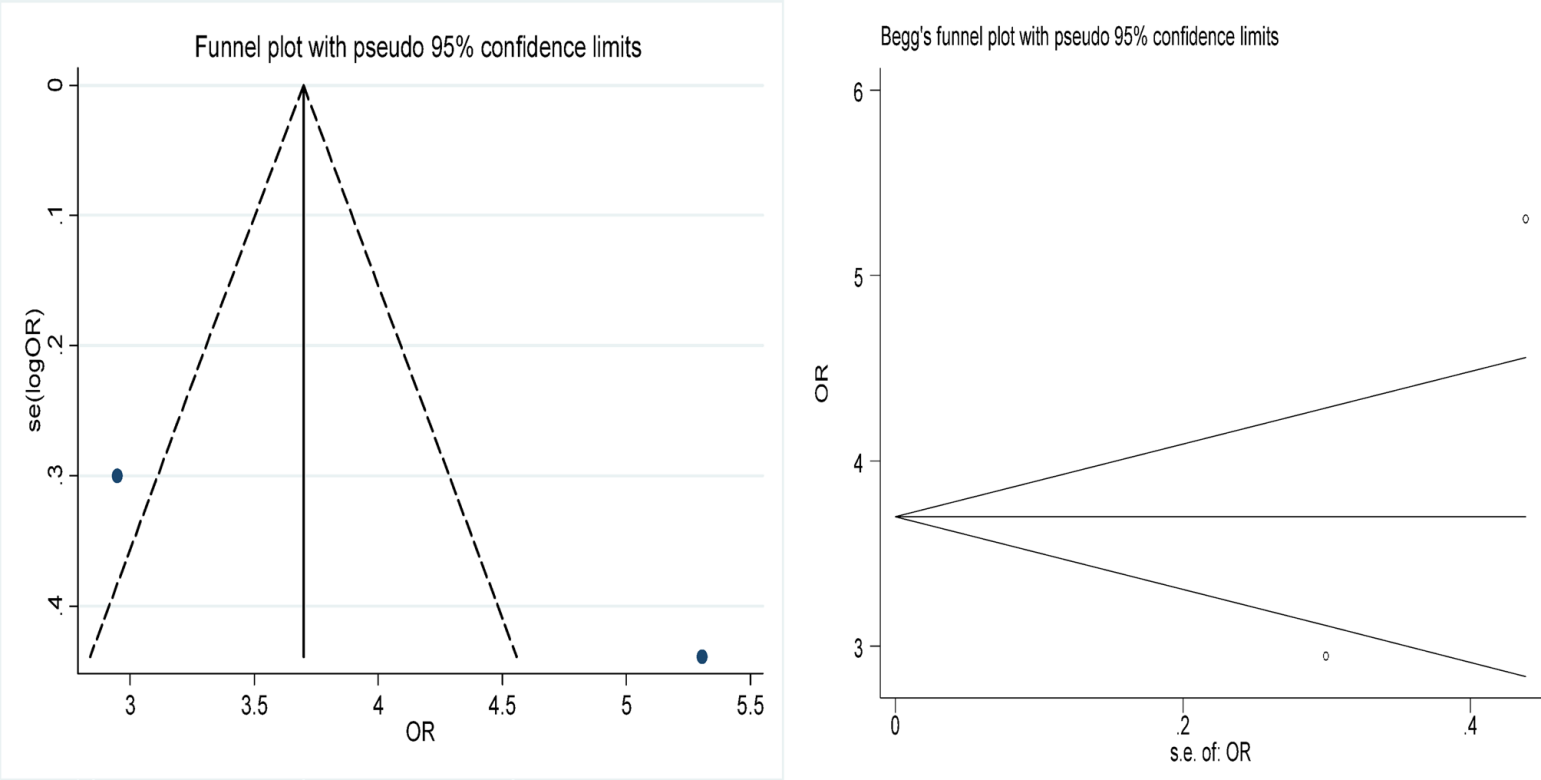
Funnel plot of difference in HPV clearance rate between the focused ultrasound group and the observation group. Begg’s Test *p* = 1.000.

**Fig. 12:**
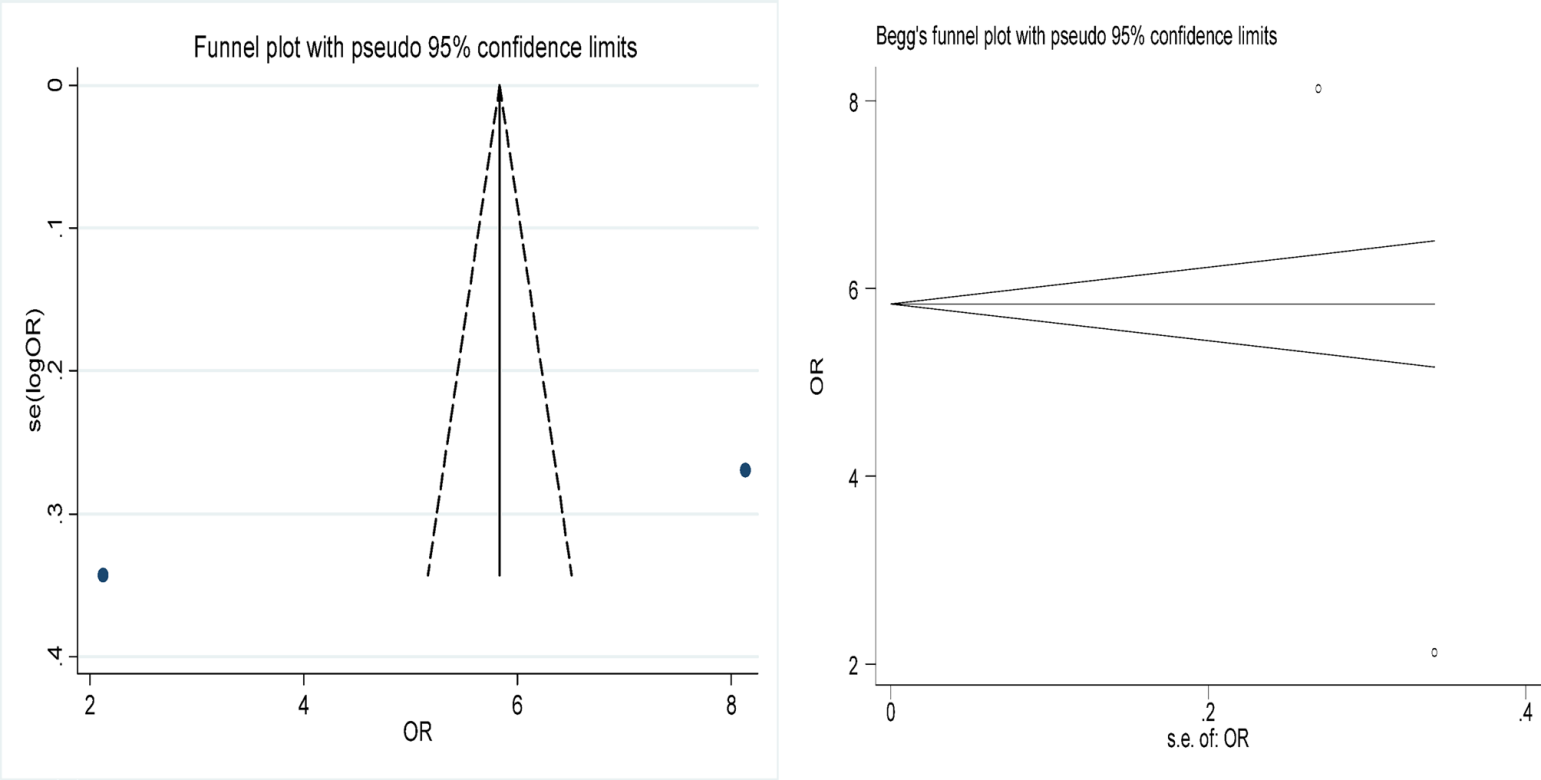
Funnel plot of difference in the clearance rate of HPV between the focused ultrasound group and interferon group. Begg’s Test *p* = 1.000.

The funnel plots of the included studies are shown in Figs. 8, 9, 10, 11 and 12, all showing roughly symmetrical shapes. In addition, we conducted the Begg’s test and the Egger’s test to evaluate the presence of publication bias in this study. No significant publication bias was found for any of the results (all *P* > 0.05).

## Discussion

Persistent high-risk HPV (hrHPV) infection is strongly associated with high-grade squamous intraepithelial lesions (HSIL) and cervical cancer, as hrHPV is detected in nearly all cases of cervical precancerous lesions and invasive disease. Consequently, effective management of HPV-related lesions is essential. Our analysis showed that focused ultrasound treatment resulted in HPV clearance in 74% of cases. Additionally, it achieved resolution rates of 94% for LSIL and 87% for abnormal ThinPrep cytology test (TCT) results. Moreover, focused ultrasound demonstrated significantly higher efficacy in clearing HPV compared to both the observation and interferon groups.

A study from China reported that among HPV-positive patients under the age of 30 with high-grade squamous intraepithelial lesions (HSIL), focused ultrasound treatment led to a higher rate of high-risk HPV (hrHPV) clearance at one-year follow-up compared to the Loop Electrosurgical Excision Procedure (LEEP). These findings suggest that focused ultrasound may be a promising treatment option for young patients with HSIL^31^. Our findings align with Xiao et al.^31^. However, unlike LEEP—which is standard for CIN2+—FU’s role in LSIL remains controversial, given that > 90% of CIN1 regresses spontaneously^17^. Thus, FU should be reserved for select cases: persistent hrHPV (> 12 months), significant patient anxiety, or desire for rapid lesion resolution prior to pregnancy.

Focused ultrasound has been shown to be more effective than cryotherapy in the treatment of cervical LSIL. However, both focused ultrasound and cryotherapy demonstrate similar efficacy in clearing HPV, with no significant differences in complication rates^26^. Additionally, one study suggests that lower vaginal microbial diversity may be associated with high-risk (HR) HPV infection. Focused ultrasound treatment may contribute to a reduction in vaginal microbial diversity and an increase in the abundance of Bifidobacterium, which could have potential implications for HPV clearance^21,32^.

This review includes observational studies, some of which report large effect sizes. As a result, the quality of evidence was upgraded by one level, though it remains rated as low to moderate. Potential confounding factors may be present in the included studies, limiting confidence in the findings. Therefore, the actual outcomes may differ from those reported, highlighting the need for more high-quality randomized controlled trials to validate these results. To our knowledge, this is the first systematic review evaluating the efficacy of focused ultrasound in the treatment of HPV and cervical lesions. The findings suggest that focused ultrasound is an effective and safe treatment option for HPV, with the additional benefit of simultaneously treating cervical LSIL and abnormal ThinPrep cytology test (TCT) results. This approach may provide a promising option for young women and clinicians managing HPV-related cervical lesions.

## Limitations

This study has several limitations. First, the meta-analysis includes only observational studies and no randomized controlled trials (RCTs), highlighting the need for more high-quality RCTs to strengthen the reliability of the findings. Second, the sample size of the included studies is relatively small, and larger studies are needed to improve the robustness of the results. Third, all the included studies originate from China, with four studies published by the same first author, which may introduce regional and author-related bias, limiting the generalizability of the findings to broader populations. Finally, this review has a high potential for bias due to substantial heterogeneity (I² = 92.3% for HPV clearance), and it is not possible to completely rule out potential confounding factors in the included studies.

## Future research requirements

Future research on the use of focused ultrasound for treating HPV and cervical LSIL should address key methodological factors. To enhance the reliability of conclusions, multicenter randomized controlled trials with larger sample sizes are needed. Additionally, there is a lack of literature on recurrence rates and potential side effects, particularly long-term obstetric safety, associated with focused ultrasound treatment for HPV and cervical LSIL. Further research should focus on these aspects to provide more comprehensive data, ultimately facilitating the integration of focused ultrasound into clinical practice. Addressing these gaps would significantly strengthen the evidence base for this treatment approach.

## Conclusion

This meta-analysis demonstrates that focused ultrasound achieves a 74% HPV clearance rate and 94% LSIL resolution in women with cervical LSIL and concurrent hrHPV infection. While superior to observation and interferon, the evidence remains low-to-moderate due to the observational nature and geographic concentration of included studies. Future multicenter RCTs are essential to validate these results and assess long-term outcomes, including recurrence and obstetric safety.

## Supplementary Information

Below is the link to the electronic supplementary material.

Supplementary Material 1

## Data Availability

All data generated or analyzed during this study are included in this published article.

## Abbreviations

HPV: Human papillomavirus
RCT: Randomized controlled trial
MINORS: Methodological Index for Non-Randomized Studies
95% CI: 95% confidence interval
LSIL: Low-grade squamous intraepithelial lesions
TCT: ThinPrep cytology test
HSIL: High-grade squamous intraepithelial lesions
CIN: Cervical intraepithelial neoplasia
FU: Focused Ultrasound
LEEP: Loop Electrosurgical Excision Procedure
