# Supplementary material for: Efficacy of focused ultrasound for HPV clearance and cervical LSIL treatment: a meta-analysis
Source: Sci Rep. 2026 Mar 28;16:10747. doi: 10.1038/s41598-026-45421-4 (PMC13039728; doi:10.1038/s41598-026-45421-4)
Supplement: Supplementary file 1 — Supplementary Material 1 [file 41598_2026_45421_MOESM1_ESM.docx]

**Identification of studies via databases and registers**

A manual search of PubMed, Embase, and Cochrane Library was performed from the date of database establishment to November 2024

**Identification**

After reading the title and abstract, 3691 studies were excluded

At the first screening, 3803 studies were obtained

**Screening**

After checking duplicate studies and reading the full text, 102 studies were excluded

112 studies were obtained

Finally, 10 studies were included

**Included**

*From:*  Page MJ, McKenzie JE, Bossuyt PM, Boutron I, Hoffmann TC, Mulrow CD, et al. The PRISMA 2020 statement: an updated guideline for reporting systematic reviews. BMJ 2021;372:n71. doi: 10.1136/bmj.n71

For more information, visit: <http://www.prisma-statement.org/>
